# Fluorescent Probe with Dual Energy Transfer Process for Selective Fe^3+^ Detection and Cellular Imaging

**DOI:** 10.3390/mi17080957

**Published:** 2026-08-13

**Authors:** Lei Zhong, Liang Chen, Fagu Zeng, Xiuji Wang, Yihua Gao

**Affiliations:** School of Pharmacy, Guangdong Medical University, Dongguan 523808, China; zhonglei1@gdmu.edu.cn (L.Z.); chenl233@gdmu.edu.cn (L.C.); zengfagu@gdmu.edu.cn (F.Z.)

**Keywords:** aggregation-induced emission, fluorescence resonance energy transfer, dark resonance energy transfer, silole, rhodamine, selectivity, Fe^3+^ detection, cell imaging

## Abstract

Traditional fluorescent probes often exhibit compromised response and specificity due to poor adaptability to varying polar environments. Herein, we present the development of a robust Fe^3+^-specific small-molecule sensor by linking a tetraphenylsilole derivative and rhodamine 6G hydrazide via a Schiff-base π bridge to form a fluorescent donor–acceptor system. The dispersed silole moiety serves as dark donor, while the aggregated state of silole converts into emissive donor. Upon selective binding with Fe^3+^, the molecules are found to undergo fluorescence resonance energy transfer (FRET) and dark resonance energy transfer (DRET) to rhodamine moiety in a polarity-dependent manner. Hence, fluorescence quantitation of Fe^3+^ in both high-organic (>70%) and water-rich solutions (>70%) is successfully achieved with detection limits of 0.083 μM and 0.28 μM, respectively. Further, ratiometric intracellular imaging of Fe^3+^ is demonstrated using the probe. This sensing strategy can offer a promising avenue for the development of polarity-adaptive fluorescent probes targeting other metal ions in complex biological and environmental matrices.

## 1. Introduction

Ferric ions (Fe^3+^) constitute one of the vital micronutrients required for human physiological function [1], as it is irreplaceable for iron storage, systemic iron transportation, and numerous key physiological process. Specifically, Fe^3+^ is required for the biosynthesis of myoglobin in muscle cells, the maintenance of immune function, the regulation of enzyme activity, hormone synthesis and erythropoiesis [2,3,4]. Notably, the abnormal fluctuations in concentration of Fe^3+^ can directly contribute to the onset and progression of various pathological conditions, including diabetes mellitus, anemia, liver injury, cancer, neurodegenerative diseases, hemochromatosis, heart failure and liver fibrosis [5,6]. Given the pivotal role of Fe^3+^ in biochemical reactions and its close association with disease pathogenesis, the establishment of analytical methodologies characterized by high sensitivity and selectivity for the real-time detection of Fe^3+^ in these complex biological matrices has attracted sustained research interest.

To date, a wide array of analytical methodologies has been developed for the determination of Fe^3+^, encompassing techniques ranging from atomic spectroscopy and mass spectrometry to electrochemical and colorimetric approaches [3,5,7,8], including atomic spectroscopy, inductively coupled plasma mass spectrometry (ICP-MS), inductively coupled plasma atomic emission spectrometry (ICP-AES), electrochemical methods, and colorimetric assays. While offering high sensitivity and accuracy, these methods generally require sophisticated instrumentation, laborious sample pretreatment, and are generally unsuitable for real-time or in situ analysis. Moreover, many of these methods are not compatible with live-cell imaging or the dynamic monitoring of Fe^3+^ fluctuations in complex environments [9]. Due to the non-invasive nature and compatibility with living systems, fluorescent probes have garnered considerable attention as alternatives for Fe^3+^ detection, such as rhodamine-based, Pyrrolo-based and thiazolothiazole-based chemosensors [10,11,12]. Nevertheless, conventional fluorophores with π-planar structures frequently suffer from aggregation-caused quenching (ACQ) in aqueous environments, false positives from non-specific interaction, and limited Stokes shifts significantly compromising signal-to-noise ratios [10,13,14].

Unlike single fluorophores, energy transfer strategies in donor–acceptor systems have attracted increasing interest for enriching the functionality and sensitivity of fluorescent probes. Among various photophysical strategies, fluorescence resonance energy transfer (FRET) serves as a prevalent and efficient approach for engineering molecular cassettes characterized by large emission shifts and significant pseudo-Stokes shifts [15,16,17,18]. The fundamental prerequisite for an efficient FRET cascade is substantial spectral overlap between the donor’s emission profile and the acceptor’s absorption band. Within these rationally designed FRET cassettes, the excitation energy is transferred through a non-radiative, dipole–dipole coupling mechanism across space, effectively bypassing radiative decay from the donor. A distinct advantage of the FRET architecture lies in its capacity for simultaneous signal acquisition at both the donor and acceptor emission channels. This dual-readout capability facilitates the self-calibrating, ratiometric quantification of target analytes, thereby minimizing environmental interference. Based on the FRET mechanism, Chang and co-workers proposed a new strategy named dark resonance energy transfer (DRET) [19,20,21]. In this mechanism, the dark donor possesses a high molar extinction coefficient and absorbs light efficiently but lacks fluorescence quantum yield due to rapid internal conversion or intersystem crossing. This dark donor strategy offers unique photophysical advantages. It eliminates the background fluorescence of the donor, making the acceptor the sole emission source, thereby significantly improving the signal-to-noise ratio.

Aggregation-induced emission (AIE), first coined by Tang and co-workers in 2001, describes a counterintuitive photophysical phenomenon where certain fluorophores are virtually non-emissive in dilute solution but become strongly fluorescent upon aggregation [22]. This behavior stands in sharp contrast to the conventional aggregation-caused quenching (ACQ) effect exhibited by most traditional fluorophores. The underlying mechanism is widely attributed to the restriction of intramolecular motion (RIM)—including rotation, twisting, and vibration—in the aggregated state, which suppresses non-radiative decay pathways and channels excitation energy into photon emission [23]. Since its discovery, AIE has evolved from a fundamental curiosity into a versatile platform with broad applications spanning materials science, analytical chemistry, and biomedical engineering [24,25,26,27].

Based on the unique photophysical duality, AIE-active luminogens are allowed to function either as fluorescent donors or dark donors within energy transfer cassettes depending on their molecular dispersion state. Several AIE-active fluorophores have been successfully implemented in the construction of FRET and DRET cassettes [28,29,30]. However, reports on their application in ion sensing remain relatively scarce. In our prior work, a fluorescent probe with polarity-sensitive FRET and DRET processes was exploited and utilized for Fe^3+^ detection across different polar environments [20]. Nevertheless, there still exists an urgent necessity to expand the diversity of accessible molecular scaffolds.

**Scheme 1:**
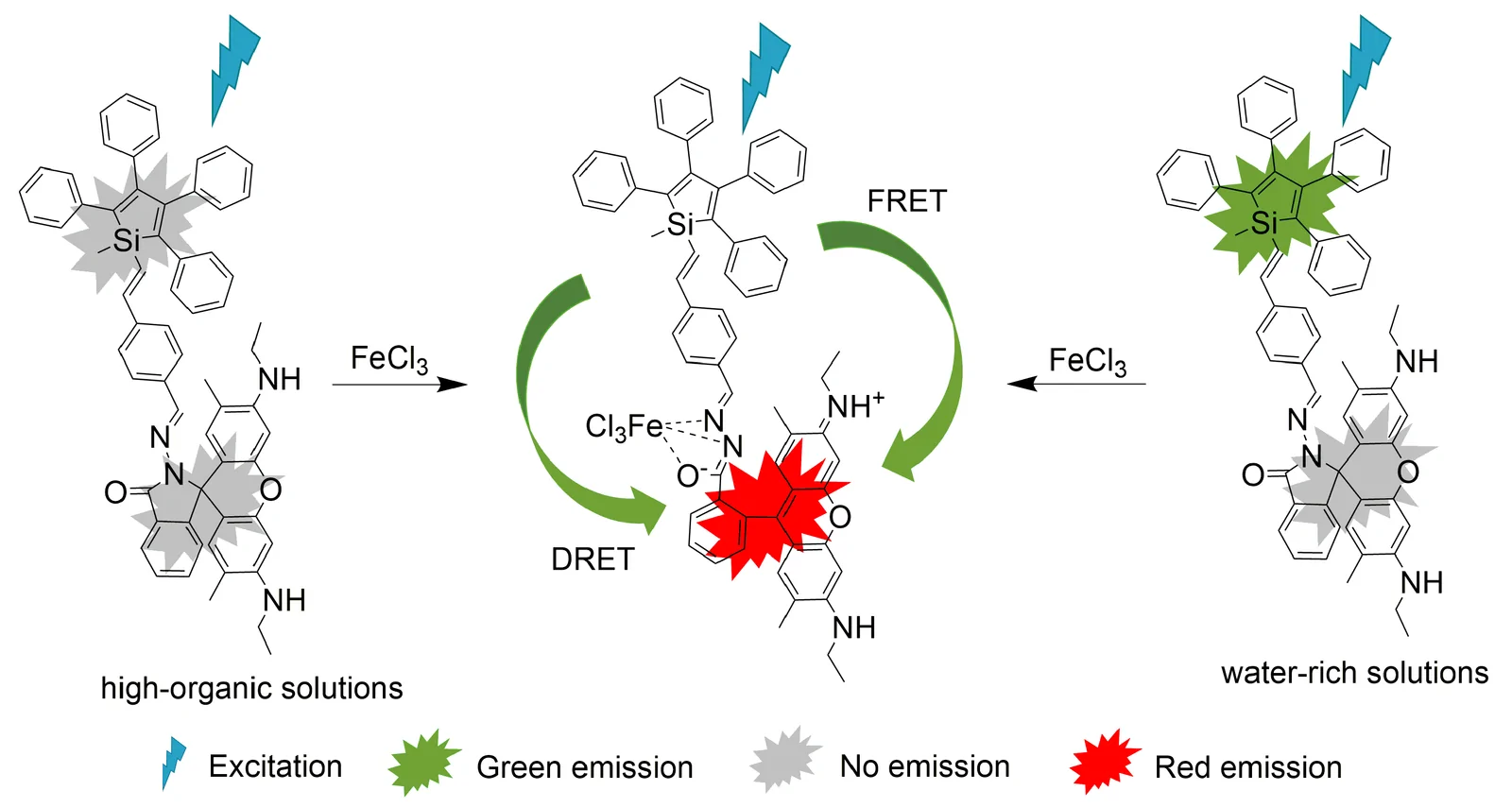
Proposed Fe^3+^ sensing mechanism of **SiRh**.

Herein, we establish a donor–acceptor fluorescent probe (**SiRh**) for Fe^3+^ sensing (Scheme 1). Distinct from our previous work employing a flexible diethylenetriamine linker, the probe integrates an AIE-active tetraphenylsilole derivative with rhodamine 6G via a rigid Schiff base bridge. This structural design is deliberatively chosen to define and regulate the through-space energy transfer pathway. While the silole unit functions as a dark donor in low-water-content solutions and a luminescent donor in high-water-content environments, the rigid Schiff base spacer fixes the donor–acceptor spatial arrangement. This structural constraint ensures that the energy transfer occurs via a predictable and well-defined through-space pathway. Notably, the Schiff base moiety is pivotal for the probe’s turn-on mechanism, as the robust multi-dentate chelation site rendered rhodamine moiety the septicity to bind with Fe^3+^ and undergo ring-opening reaction at the spirolactam. This structural transformation induces a significant red shift in the acceptor’s absorption, optimizing the spectral overlap with the silole donor. Critically, the polarity-dependent aggregation of the silole core dictates the energy transfer pathway: in aqueous-rich environments, the aggregated silole functions as a fluorescent donor, facilitating standard FRET, whereas in organic-rich media, the monomeric silole acts as a dark donor, initiating the DRET process. As a result, polarity-adaptive and specific detection of Fe^3+^ was achieved with a pseudo large Stokes shift of 192 nm, which was further demonstrated for cellular imaging.

## 2. Results and Discussion

### 2.1. Structural Characterization and Spectral Properties of Probe SiRh

**Scheme 2:**
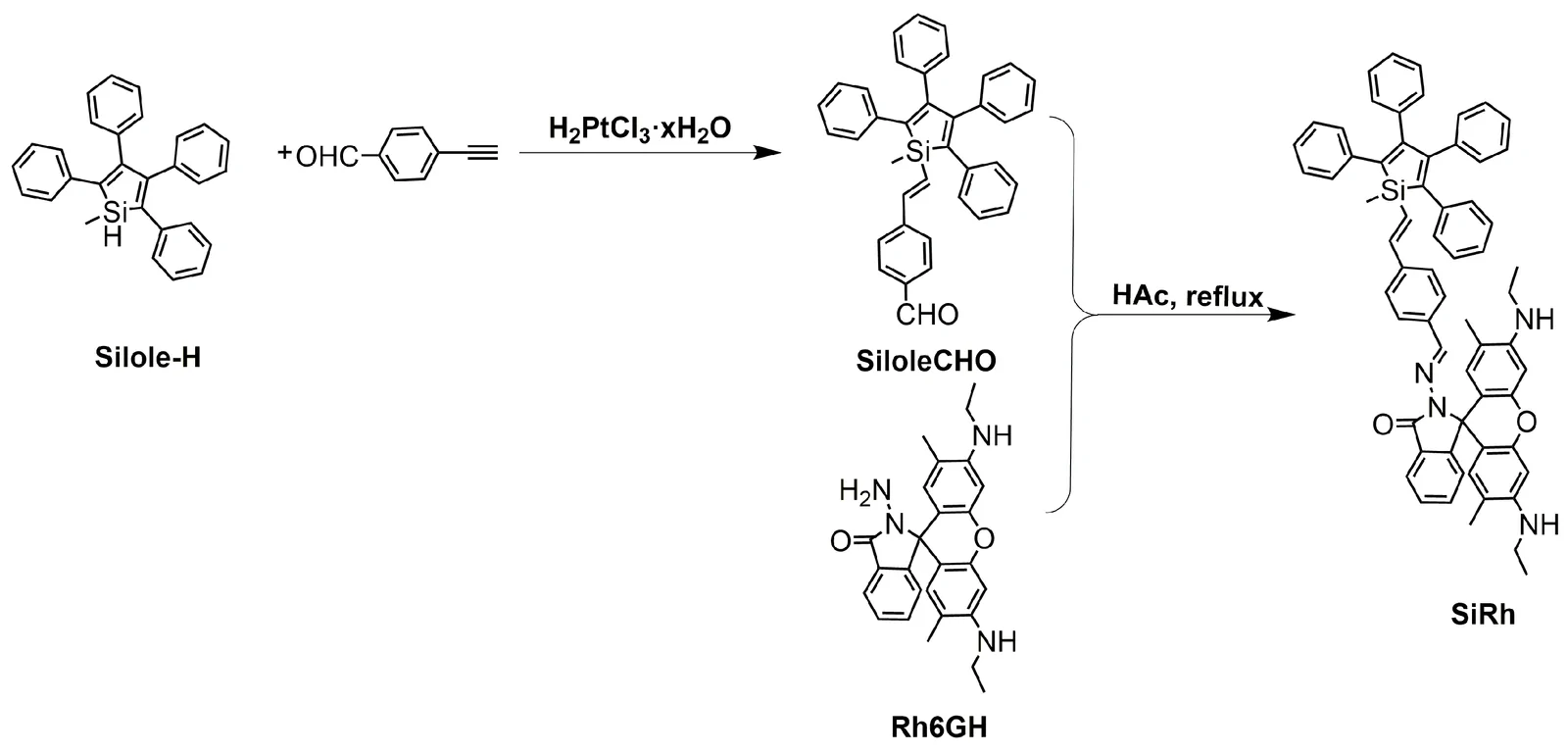
Synthetic route of **SiRh**.

The chemical structure and synthetic pathway of **SiRh** are presented in Scheme 2, which was corroborated via a combination of spectroscopic and mass spectrometric techniques (Figures S1–S6). To clarify the AIE property of **SiRh** and lay a foundation for investigating its energy transfer mechanisms during Fe^3+^ detection, we performed fluorescence spectral measurements in tetrahydrofuran (THF)–H_2_O mixtures with varying water fractions (Figure S7). We observed that the fluorescent intensity of **SiRh** at 480 nm (ascribed to the emission of the silole moiety) was completely quenched at an H_2_O content below 70%. This was attributed to the fact that, in good solvents, the intramolecular rotations of the silole core and peripheral phenyl rings were largely unrestricted. This dynamic motion consumed the excitation energy through non-radiative decay pathways, resulting in a dark state. When the H_2_O fraction reached 70%, the fluorescence intensity at 480 nm increased significantly, rose continuously with increasing H_2_O content, and reached its maximum at 80%. These results suggest that the silole moiety of **SiRh** could serve as a dark energy donor in low-H_2_O environments, whereas **SiRh** aggregated when water content exceeded 70%, triggering fluorescence emission of the silole moiety via restricted molecular motion. In this regard, THF/H_2_O volume ratios of 2:8 and 8:2 were employed for further investigating the FRET and DRET process of **SiRh** during Fe^3+^ sensing, respectively.

### 2.2. Sensing Performance Mechanism in High-Organic Phase

**Figure 1:**
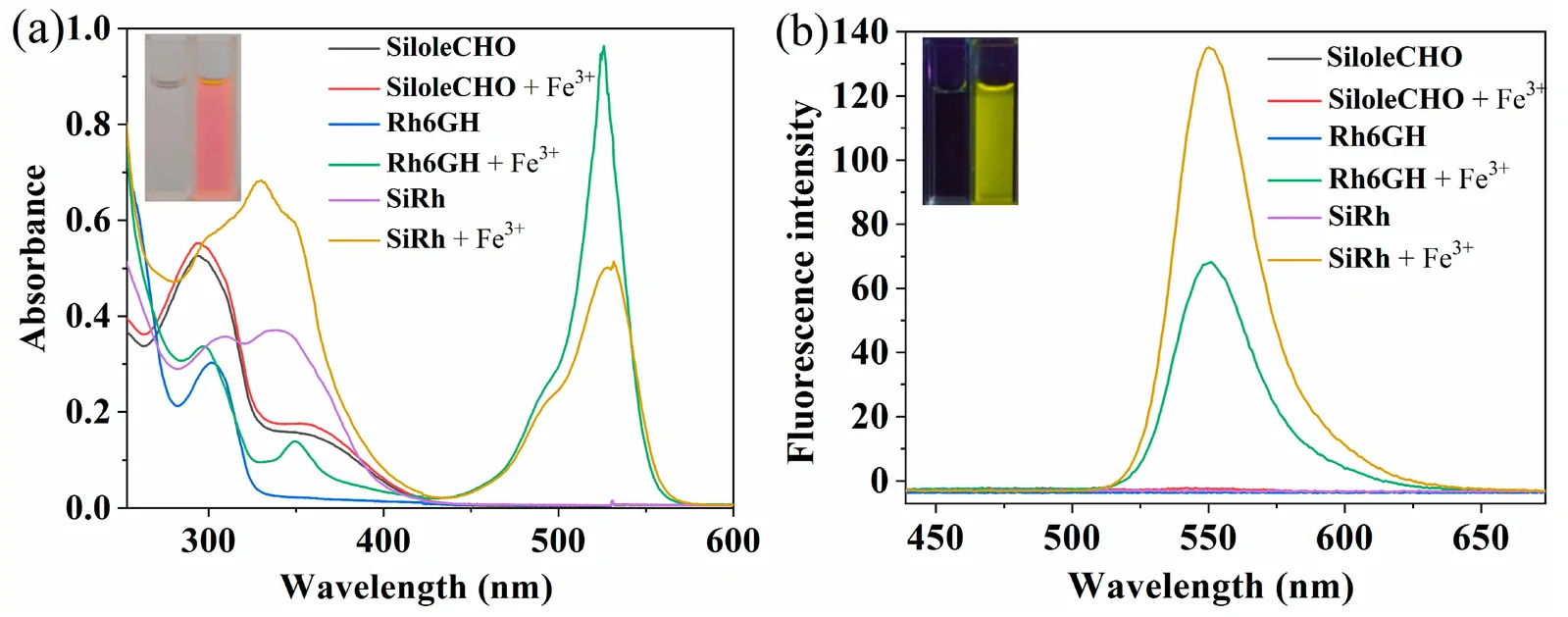
Spectral responses of **SiRh** and its precursors to Fe^3+^ in high-organic solution. (**a**) Absorption spectra in THF/H_2_O (*v*/*v* = 8/2) with and without 2 μM Fe^3+^. (**b**) Fluorescence spectra of the above compounds under identical conditions. Insets: Corresponding photographs of **SiRh** displaying the visible color (**a**) and fluorescence (**b**) changes induced by Fe^3+^. Organic compound concentration: 10 μM; Excitation wavelength: 360 nm.

To explore the interaction between the fluorescent probe **SiRh** and Fe^3+^ ions, we next measured the ultraviolet-visible (UV–vis) absorption spectra of the donor precursor **SiloleCHO**, acceptor precursor **Rh6GH**, and probe **SiRh** in THF/H_2_O solution (*v*/*v* = 8/2) both with and without Fe^3+^. As shown in Figure 1a, **SiRh** exhibited two absorption peaks at 360 nm and 306 nm in the absence of Fe^3+^. These peaks were similar to the characteristic absorption peaks of **SiloleCHO** (363 nm) and **Rh6GH** (303 nm), suggesting that the silole moiety and the rhodamine fluorophore are independent in this dual-chromophore system. Upon the addition of Fe^3+^, the **SiRh** solution turned pink, and a novel absorption band emerged at 528 nm in the visible region. This spectral behavior parallels that of **Rh6GH**, where Fe^3+^ triggered a characteristic peak at 526 nm attributable to the ring-opened amide form of the rhodamine scaffold [10]. These observations confirm that **SiRh** is capable of functioning as a reliable chromogenic probe for Fe^3+^ sensing.

Motivated by the optical changes, the fluorescent behavior of **SiRh** upon analyte interaction was examined. The fluorescence spectra of **SiloleCHO**, **Rh6GH**, and **SiRh** were recorded in a THF/H_2_O (8:2, *v*/*v*) system with and without 2 μM Fe^3+^ (Figure 1b). Owing to its AIE feature, **SiloleCHO** displayed weak emission at 360 nm in this organic-rich medium. The emission intensity of the silole unit in **SiRh** was comparable to that of **SiloleCHO**, indicating that this unit acted as a dark donor with low intrinsic luminescence. Upon interaction with Fe^3+^, **SiRh** showed a distinct new fluorescence peak at 552 nm, which was consistent with that of Fe^3+^-treated **Rh6GH** and assigned to the characteristic emission of the ring-opened rhodamine derivative. Additionally, **SiRh** emitted orange fluorescence under UV irradiation. These results confirmed that the binding of **SiRh** to Fe^3+^ induced the spirocycle ring-opening of rhodamine and promoted efficient energy transfer from the silole dark donor to the emissive rhodamine unit.

Motivated by the optical changes, the fluorescent behavior of **SiRh** upon analyte interaction was examined. The fluorescence spectra of **SiloleCHO**, **Rh6GH**, and **SiRh** were recorded in a THF/H_2_O (8:2, *v*/*v*) system with and without 2 μM Fe^3+^ (Figure 1b). Owing to its AIE feature, **SiloleCHO** displayed weak emission at 360 nm in this organic-rich medium. The emission intensity of the silole unit in **SiRh** was comparable to that of **SiloleCHO**. As a typical AIE-active luminogen, the silole donor exhibited intense intramolecular rotations in its molecularly dissolved state. These rotations provided a dominant non-radiative decay pathway that effectively quenched the donor fluorescence. In addition, the rhodamine 6G acceptor existed predominantly in the non-emissive ring-closed spirolactam form. This dual quenching mechanism, which combines the restriction of intramolecular rotation process of the AIEgen with the intrinsic dark state of the spirolactam ring, ensured an exceptionally low background signal for the probe prior to analyte binding. Upon interaction with Fe^3+^, **SiRh** showed a distinct new fluorescence peak at 552 nm, which was consistent with that of Fe^3+^-treated **Rh6GH** and assigned to the characteristic emission of the ring-opened rhodamine derivative. Additionally, **SiRh** emitted orange fluorescence under UV irradiation. These results confirmed that the binding of **SiRh** to Fe^3+^ induced the spirocycle ring-opening of rhodamine and promoted efficient energy transfer from the silole dark donor to the emissive rhodamine unit. Crucially, while Fe^3+^ is typically a potent photoinduced electron transfer quencher, the dramatic emission enhancement driven by the ring-opening overwhelmingly eclipses any minor quenching pathways.

**Figure 2:**
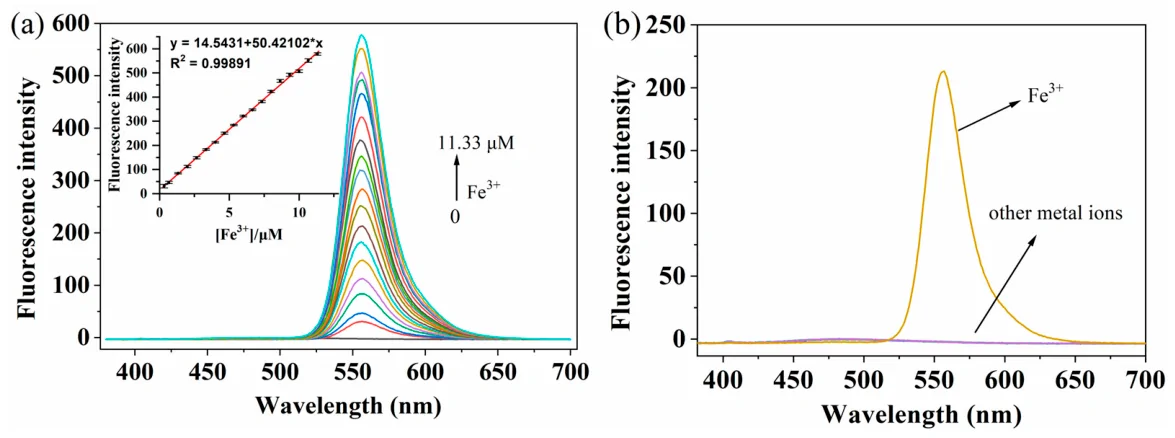
Fluorescence-sensing performance of probe SiRh toward Fe^3+^ in high-organic solution. (**a**) Emission spectra of **SiRh** in THF/H_2_O (8:2, *v*/*v*) upon addition of different concentrations of Fe^3+^ (λ_ex_ = 360 nm). The curves in different colors from bottom-to-top represent the progressively increasing concentrations of Fe^3+^. Inset: Dependence of emission intensity at 552 nm on Fe^3+^ concentration. Error bars represent standard deviation (SD) from triplicate measurements. The asterisk (*) denotes a multiplication symbol. (**b**) Emission spectra of **SiRh** in THF/H_2_O (8:2, *v*/*v*) in the presence of various metal ions. The concentration of **SiRh** was 10 μM, and the concentrations of Fe^3+^, Mg^2+^, Ca^2+^, K^+^, Na^+^, Co^2+^, Fe^2+^, Mn^2+^, Ni^2+^, Pb^2+^, Zn^2+^, Cu^2+^, Ag^+^, Hg^2+^, and Cr^3+^ were 4 μM.

The fluorescence response of **SiRh** to various Fe^3+^ concentrations in high-organic solution was further determined through fluorescence titration experiments. The emission intensity of the acceptor showed a good linear relationship with Fe^3+^ concentrations in the range of 0.33–11.33 μM (Figure 2a), and the corresponding detection limit was calculated as 0.083 μM (Table S1). While this value is higher than that of our previously reported diethylenetriamine-linked probe (0.036 μM) [6], the difference is rationally attributed to the increased steric hindrance imposed by the rigid Schiff base bridge. Unlike the flexible alkyl chain in [6], which allows Fe^3+^ ions relatively free access to the binding pocket, the planar and rigid structure of the Schiff base creates a more geometrically constrained coordination environment. Since other metal ions might interfere with rhodamine-based recognition systems, the ion selectivity of **SiRh** was also evaluated. As shown in Figure 2b, only Fe^3+^ induced a remarkable fluorescence change of **SiRh** under the experimental conditions. In stark contrast, Fe^2+^ failed to trigger such a response, primarily due to its lower charge density and weaker Lewis acidity compared to Fe^3+^. The diminished electrostatic interaction impedes the formation of a stable coordination complex with the probe’s binding sites, while other metal ions gave negligible responses. Together with the data for other metal ions, these findings demonstrated that **SiRh** exhibited favorable linear response and high selectivity toward Fe^3+^.

**Figure 3:**
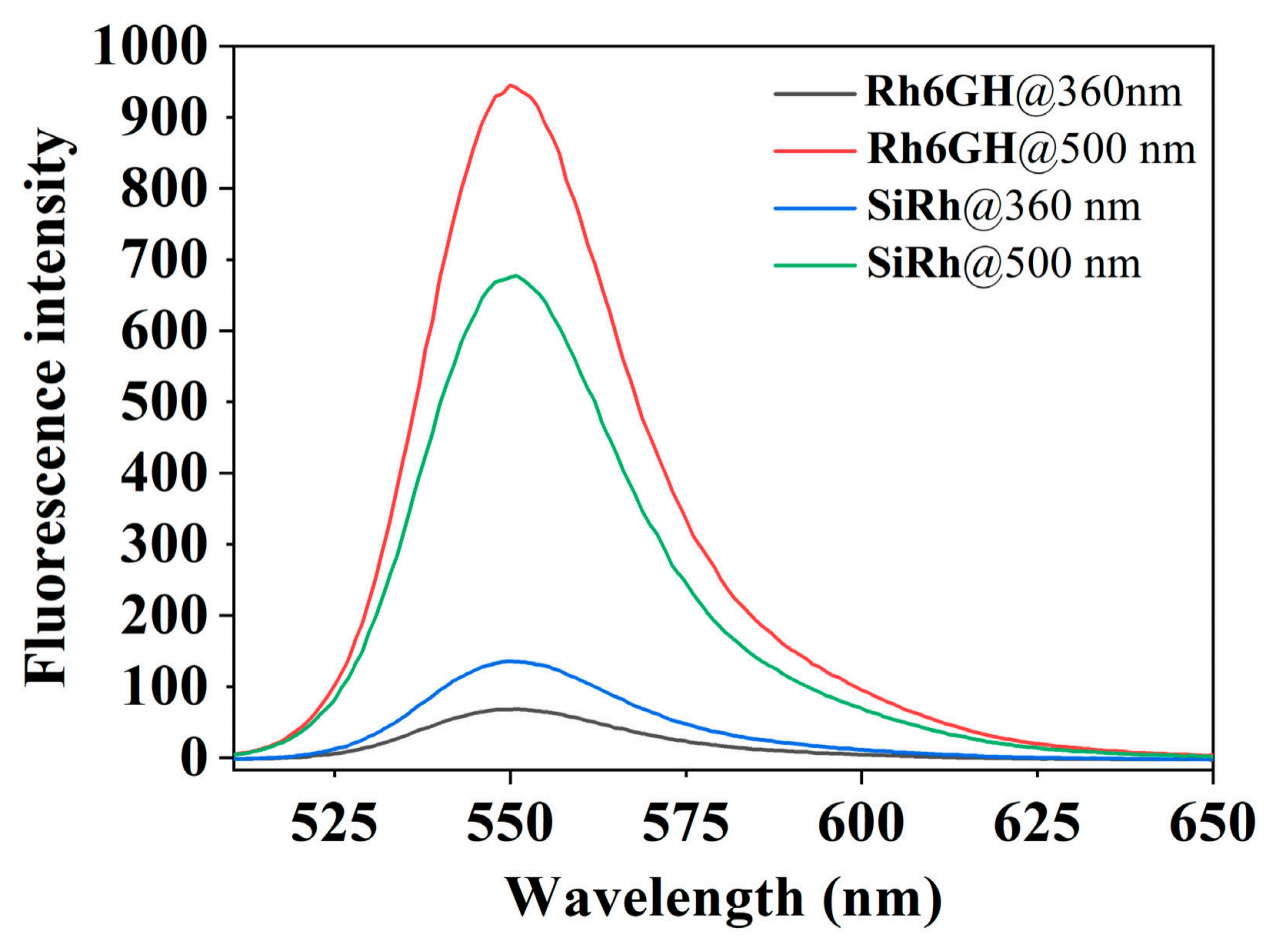
Fluorescence emission spectra of **Rh6GH** and **SiRh** upon excitation at 360 nm and 500 nm in THF/H_2_O (8:2, *v*/*v*) solution containing 2 μM Fe^3+^.

Additionally, the energy transfer behavior of the probe system in high-organic solution was also elucidated by recording the fluorescence spectra of **Rh6GH** and **SiRh** at varying excitation wavelengths (Figure 3). Under excitation at 500 nm, the absorption wavelength of Fe^3+^-bound rhodamine derivative, both **Rh6GH** and **SiRh** emitted intense fluorescence at 552 nm. When excitation shifted to 360 nm, corresponding to the absorption wavelength of the silole donor, **SiRh** displayed a significantly stronger fluorescence emission at 552 nm than **Rh6GH**. These results confirmed that the excited silole donor could efficiently transfer energy to the ground-state rhodamine acceptor via the DRET mechanism prior to non-radiative decay. With the absorption maximum at 360 nm, the probe displayed a pseudo-Stokes shift of 192 nm.

### 2.3. Sensing Performance and Mechanism in High-Water Phase

**Figure 4:**
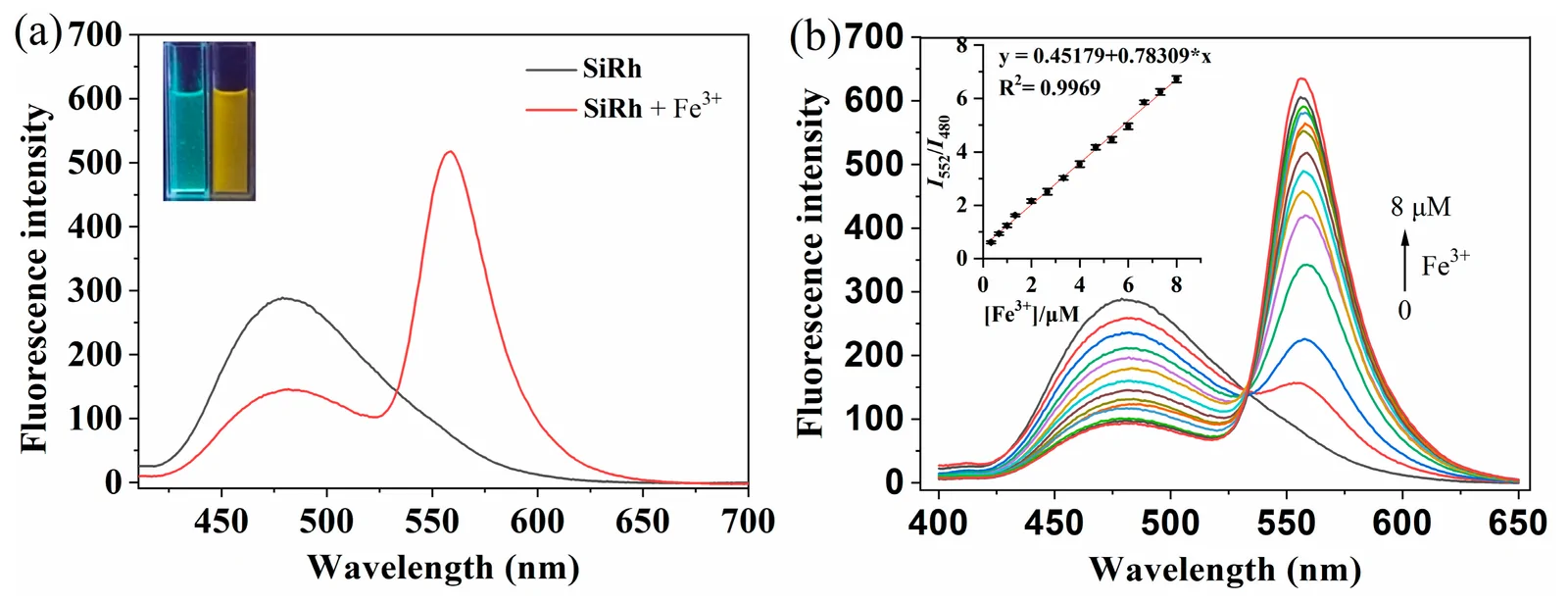
Fluorescence response of the **SiRh** probe toward Fe^3+^ in water-rich solution. (**a**) Fluorescence emission spectra of **SiRh** in THF/H_2_O (2:8, *v*/*v*) with and without 4 μM Fe^3+^. The curves in different colors from bottom-to-top represent the progressively increasing concentrations of Fe^3+^. Insets: Corresponding photographs of **SiRh** displaying the fluorescence change induced by Fe^3+^. The asterisk (*) denotes a multiplication symbol. (**b**) The fluorescence intensity ratio (*I*_552_/*I*_480_) of **SiRh** as a function of Fe^3+^ concentration. The excitation wavelength was set at 360 nm, and the probe concentration was 10 μM.

Since the applicability of fluorescence probes for biosensing and bioimaging usually demands compatibility with highly polar, high-water-content medium, we next assessed the fluorescence performance of **SiRh** in such environments. We employed a THF/H_2_O (2:8, *v*/*v*) mixture as the detection medium and excited the probe at 360 nm. The free **SiRh** probe displayed a characteristic emission peak at 480 nm, consistent with the photoluminescence properties of siloe derivatives. This emission arose from the aggregate formation of **SiRh**, in line with its AIE feature (Figure 4a). Upon binding with Fe^3+^, the emission intensity at 480 nm decreased significantly, accompanied by the appearance of a distinct emission peak at 552 nm ascribed to the acceptor fluorophore, and the solution exhibited orange fluorescence under UV illumination. These synchronous fluorescence variations of the donor and acceptor verified that the binding interaction between **SiRh** and Fe^3+^ triggered highly efficient FRET between the two fluorophore moieties.

The feasibility of quantitative Fe^3+^ detection in high-water-content medium was further validated via fluorescence titration assays (Figure 4b). Upon gradual increases in Fe^3+^ concentrations, the emission intensity at 552 nm (acceptor) increased progressively, whereas the emission intensity at 480 nm (donor) decreased gradually. Linear regression analysis of the intensity ratio *I*_552_/*I*_480_ versus Fe^3+^ concentration afforded a satisfactory linear response. The probe showed a linear detection range of 0.33–8 μM, with a calculated limit of detection of 0.28 μM (Table S1). A comparable marginal elevation was observed relative to the 0.23 μM value of the flexible-linked analogue in [6]. This cross-regime consistency stems from the shared steric constraint imposed by the rigid Schiff base on Fe^3+^ coordination, as elaborated for the DRET process above. Notably, the intensity ratio *I*_552_/*I*_480_ was enhanced by 6.8-fold upon the addition of 2 μM Fe^3+^. These results demonstrate that the **SiRh** probe could function as a highly sensitive ratiometric fluorescent probe for Fe^3+^ in water-rich solution.

**Figure 5:**
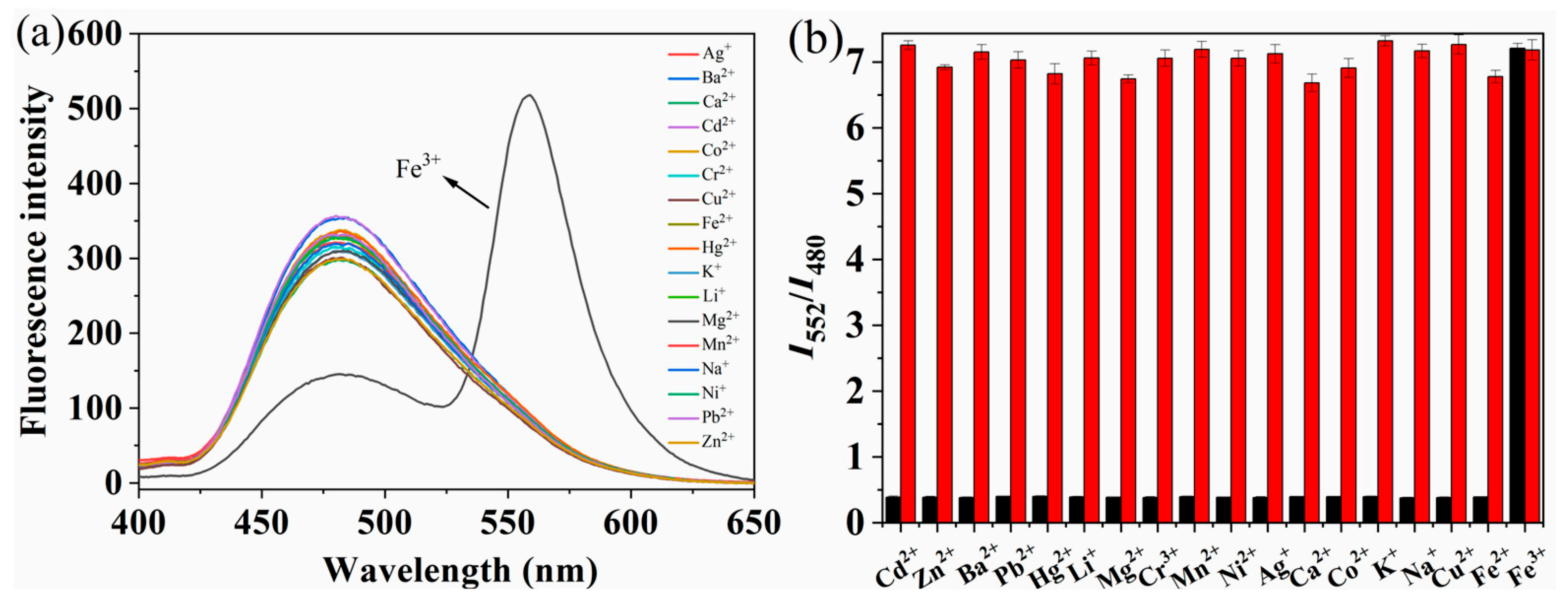
Selectivity and anti-interference of **SiRh** for Fe^3+^ in water-rich media. (**a**) Emission spectra of 10 μM **SiRh** in THF/H_2_O (2:8, *v*/*v*) with various metal ions (4 μM each). Excitation wavelength: 360 nm. (**b**) Bar chart of fluorescence intensity ratio *I*_552_/*I*_480_ before (Black bars) and after (Red bars) the addition of 4 μM Fe^3+^ into each metal-ion-containing solution (8 μM each).

To assess the ion selectivity of **SiRh**, we performed fluorescence spectroscopic tests towards various metal ions in THF/H_2_O (2:8, *v*/*v*) media (Figure 5a). Only Fe^3+^ induced a remarkable fluorescence emission shift of **SiRh** from 480 nm to 552 nm. None of the other competing metal ions elicited analogous fluorescence responses or changed the *I*_552_/*I*_480_ fluorescence intensity ratio, which verified the specific fluorescence response of **SiRh** toward Fe^3+^. We also carried out competitive experiments to evaluate the effects of coexisting metal cations on Fe^3+^ detection (Figure 5b). The Fe^3+^-induced *I*_552_/*I*_480_ fluorescence ratio remained nearly constant even in the presence of other metal ions. These results validated the outstanding selectivity and anti-interference performance of **SiRh** for ratiometric fluorescence sensing of Fe^3+^.

Electrospray ionization mass spectrometry (ESI-MS) was further utilized to explore the binding mechanism between **SiRh** and Fe^3+^ in THF/H_2_O (2:8, *v*/*v*) solution (Figure S8). A peak at the mass-to-charge ratio (*m*/*z*) of 941.42 was observed, corresponding to the protonated molecular ion peak (M + 1) of **SiRh**. Another peak at the *m*/*z* of 1242.6 was assigned to the cation [**SiRh** + FeCl_3_ + 2H_2_O + 2MeOH + CH_3_CN + H]^+^, which confirmed the formation of a 1:1 complex between **SiRh** and Fe^3+^. Consistent mass spectral profiles were also obtained in the THF/H_2_O (8:2, *v*/*v*) media (Figure S9). In addition, Job’s plot experiments were performed to further verify the 1:1 binding stoichiometry between **SiRh** and Fe^3+^ (Figure S10). The binding constant (K_a_) was determined to be 1.5 × 10^3^ L/mol (THF/H_2_O = 8/2, *v*/*v*) and 4.3 × 10^4^ L/mol (THF/H_2_O = 2/8, *v*/*v*) by fluorescence titration using the Benesi–Hildebrand equation. To gain deeper insight into the coordination-induced structural changes, Fourier Transform Infrared Spectroscopy (FT-IR) was employed (Figure S11). In the spectrum of free **SiRh**, the characteristic absorption bands of the closed spirolactam form were observed at 1714 cm^−1^ (amide C=O stretch) and 1622 cm^−1^ (imine C=N stretch). Upon addition of Fe^3+^, the C=O stretching band exhibited a significant red shift to 1651 cm^−1^, accompanied by a decrease in intensity, and the C=N stretching band shifted to 1606 cm^−1^. These red shifts resulted from the disruption of the spirolactam ring and the delocalization of electrons into the conjugated xanthene system upon metal coordination. On this basis, the sensing mechanism of **SiRh** towards Fe^3+^ was proposed (Scheme 1). In Fe^3+^-free THF/H_2_O (8:2, *v*/*v*) solution, the silole moiety exhibited weak fluorescence as a dark donor, whereas it underwent aggregation and acted as luminescent donor with enhanced fluorescence in THF/H_2_O (2:8, *v*/*v*) media. Upon coordination with Fe^3+^ ions, the rhodamine moiety underwent spirolactam ring opening and displayed a red shift in its absorption spectrum (Figure 1a). The increased spectral overlap between the rhodamine and silole moieties facilitated energy transfer through FRET and DRET process, which was depended on solvent polarity. Eventually, characteristic fluorescence emission from rhodamine moiety was observed.

### 2.4. Intracellular Fe^3+^ Imaging

**Figure 6:**
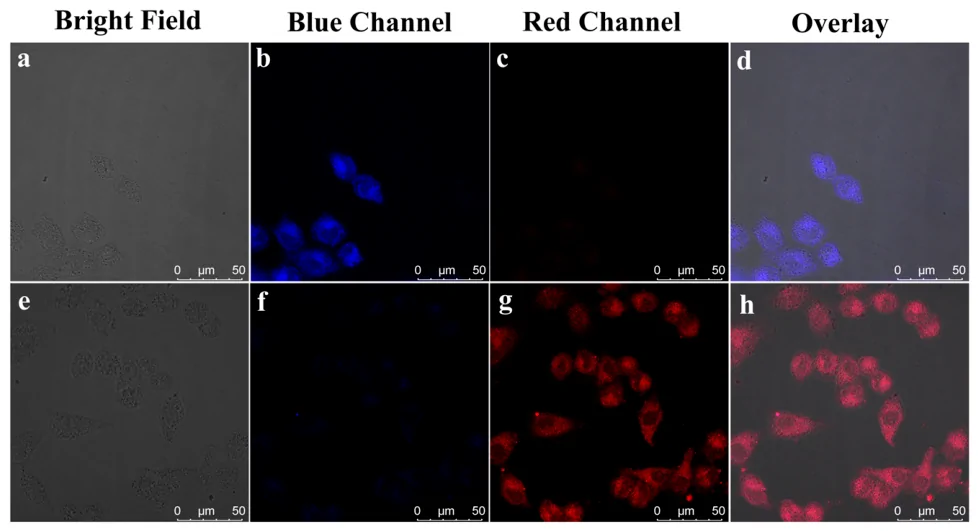
CLSM imaging of SiRh-mediated Fe^3+^ detection in HeLa cells. HeLa cells were incubated with 10 μM **SiRh** for 1 h, followed by 20 min incubation in the absence (**a**–**d**) or presence (**e**–**h**) of 20 μM Fe^3+^. (**a**,**e**) Brightfield images. (**b**,**f**) Blue channel images (emission: 450–510 nm). (**c**,**g**) Red channel images (emission: 550–610 nm). (**d**,**h**) Overlay images. Excitation wavelength: 405 nm.

Given the outstanding sensing performance of **SiRh** toward Fe^3+^ in solution, we further investigated its potential for bioimaging applications using confocal laser scanning microscopy (CLSM) (KEYENCE, Singapore). In the absence of Fe^3+^, Hela cells treated with **SiRh** alone exhibited strong intracellular blue fluorescence from the silole donor, while red fluorescence signal was barely detectable (Figure 6a–d), confirming the good cell permeability of **SiRh**. Following the addition of 20 μM Fe^3+^ to the cell culture medium, the blue fluorescence was significantly quenched, and the red channel fluorescence intensity was markedly enhanced (Figure 6e–h). Moreover, we examined the interference of common metal ions including Cu^2+^, Zn^2+^, Mg^2+^ and Ca^2+^ on **SiRh**-mediated Fe^3+^ detection in living cells (Figure S12). These cations caused no significant interference with the binding between **SiRh** and Fe^3+^, even at high concentrations. The cytotoxicity of **SiRh** was also assessed via the MTT assay (Figure S13), which showed no obvious effect on HeLa cell viability at 50 μM. Collectively, these results confirmed that **SiRh** is a highly selective and efficient fluorescent probe for Fe^3+^ imaging in living cells.

## 3. Materials and Methods

All commercially available reagents were used as received without further purification. Details regarding the commercial sources of reagents, instruments, and software used in this study are provided in the Supporting Information. Compound **SiloleCHO** and **SiRh** were synthesized as follows.

Synthesis of **SiloleCHO:** 1 g (2.6 mmol) of **Silole-H** [6] and 0.41 g (3.15 mmol) of p-Ethynylbenzaldehyde were placed into a 50 mL three-neck round-bottom flask. The reactants were dissolved in 20 mL of toluene (PhMe) under an argon atmosphere, followed by the addition of a catalytic amount of H_2_PtCl_6_·xH_2_O. The reaction was conducted at 80 °C for a duration of two days. Following this period, the reaction mixture was spotted on a chromatographic plate using a hexane/ethyl acetate (EtOAc) solvent system in a 10:1 ratio. Subsequently, column chromatography was performed using a hexane/EtOAc ratio of 20:1 to purify the product. The desired compound **SiloleCHO** was obtained by collecting the second spot. The crude product was then evaporated to dryness, washed with diethyl ether, and filtered, yielding a yellow solid at a yield of 76%. ^1^H NMR (600 MHz, DMSO-d_6_), δ (ppm): 10.00 (s, 1H, CHO), 7.88 (d, 2H, *J* = 12 Hz, ArH), 7.76 (d, 2H, *J* = 12 Hz, HC=CH), 7.12-6.80 (m, 22H, ArH), 0.65 (s, 3H, CH_3_). ^13^C NMR (150 MHz, DMSO-d_6_), δ (ppm): 192.59, 155.03, 146.84, 142.62, 139.64, 138.87, 138.46, 135.93, 129.96, 129.39, 128.50, 128.08, 127.63, 127.41, 126.55, 125.87. ESI-MS calculated for M^+^, C_38_H_30_OSi, 530.2066. Found 545.2299 [M+CH_3_]^+^.

Synthesis of **SiRH:** In a 100 mL single-neck round-bottom flask, 0.33 mmol (0.177 g) of **siloleCHO** and 0.36 mmol (0.157 g) of Rhodamine 6G hydrazide (**Rh6GH**) were combined. A total of 60 mL of anhydrous ethanol were added to dissolve the reactants, followed by the addition of 3 drops of acetic acid. The mixture was then subjected to reflux for a duration of one night. Subsequently, a column chromatography was performed using a hexane/ethyl acetate (EtOAc) solvent system with a 10:1 ratio for elution. The desired product was isolated by collecting the second spot using a hexane/EtOAc ratio of 50:1. The crude product was then evaporated to dryness, washed with diethyl ether, and filtered, yielding a yellow solid at a yield of 45%. ^1^H NMR (600 MHz, DMSO-d_6_), δ (ppm): 8.58 (s,1H, N=CH), 7.91 (d, 1H, HC=CH), 7.61-7.48 (m, 4H, ArH), 7.35 (d, 1H, *J* = 12 Hz, CH=CH), 7.14-6.71 (m, 24H, ArH), 6.33 (s, 2H, ArH), 6.16 (s, 2H, ArH), 5.05 (t, 2H, *J* = 6 Hz, NHCH_2_), 3.15-3.09 (m, 4H, NHCH_2_), 1.82 (s, 6H, CCH_3_), 1.19 (t, 6H, *J* = 12 Hz, CH_2_CH_3_), 0.61 (s, 3H, SiCH_3_). ^13^C NMR (150 MHz, DMSO-d_6_), δ (ppm): 164.26, 155.31, 151.95, 151.40, 148.31, 147.81, 146.09, 140.25, 139.40, 139.18, 138.96, 135.24, 134.40, 129.87, 128.94, 128.51, 128.07, 127.49, 127.30, 126.97, 126.27, 124.27, 123.69, 123.50, 118.74, 105.33, 96.18, 65.96, 37.93, 17.45, 14.62, 6.05. ESI-MS calculated for M^+^, C_64_H_56_N_4_O_2_Si, 940.4173. Found 941.4268 [M+1]^+^.

## 4. Conclusions

In this work, a novel donor-π-acceptor fluorescent probe featuring a large Stokes shift was constructed for the selective detection of Fe^3+^. Distinct from conventional probes relying on a single energy transfer mechanism, typically plagued by inadequate adaptability to solvent polarity variations, the proposed probe integrated FRET and DRET strategies to enable Fe^3+^ sensing in mixed THF/H_2_O solvents with varying polarities. An AIE-active silole chromophore was employed as a dual-function energy donor. In media with low water fraction (less polar), it acted as a dark donor to initiate the DRET process. In contrast, it aggregated into a luminescent donor to mediate FRET in systems with high water fraction (more polar). Both pathways yielded distinct fluorescence emission profiles from the rhodamine-based acceptor. Systematic characterization and evaluation verified that the probe exhibited excellent selectivity and sensitivity for Fe^3+^. CLSM imaging experiments further validated its utility as a high-performance ratiometric agent for Fe^3+^ detection in living cells. Therefore, this study presents a versatile design strategy for Fe^3+^ sensing in multi-polarity environments, holding great promise for applications in complex chemical and biological systems.

## Data Availability

The data presented in this study are available upon request from the corresponding author.

## Supplementary Materials

The following supporting information can be downloaded at: https://www.mdpi.com/article/10.3390/mi17080957/s1. Figure S1: ^1^H NMR of compound SiloleCHO (600 MHz, DMSO). Figure S2: ^13^C NMR of compound SiloleCHO (150 MHz, DMSO-d6). Figure S3: ESI-MS spectra of SiloleCHO. Figure S4: ^1^H NMR of compound SiRh (600 MHz, DMSO-d6). Figure S5: ^13^C NMR of compound SiRh (150 MHz, DMSO-d6). Figure S6: HRMS spectra of SiRh. Figure S7: Fluorescence spectra of SiRh (10 μM) in THF/H_2_O mixtures with different water fractions. Excitation was performed at 360 nm. Inset: Fluorescence intensity at 480 nm as a function of water fraction. Figure S8: ESI-MS spectra of the mixture of SiRh (10 μM) and Fe^3+^ (20 μM) in THF/H_2_O solution (2/8, *v*/*v*). Figure S9: ESI-MS spectra of the mixture of SiRh (10 μM) and Fe^3+^ (20 μM) in THF/H_2_O (8:2, *v*/*v*). Figure S10: Job’s plot of SiRh and Fe^3+^ in THF/H_2_O solution (8/2, *v*/*v*). The total concentration of SiRh and Fe^3+^ was 50 μM. Detection wavelength was set at 530 nm. Figure S11: Partial IR spectra of SiRh in the absence and presence of Fe^3+^. Figure S12: Fluorescence images of HeLa cells incubated with SiRh (10 μM) for 1 h followed by 20 min of incubation with Fe^3+^ (20 μM) in the absence and presence of coexisting ions (Cu^2+^, Zn^2+^, 200 μM; Mg^2+^, Ca^2+^, 1 uM). (a, e, i, m, q) Bright field images. (b, f, j, n, r) Blue channel images: 450–510 nm. (c, g, k, o, s) Red channel images: 550–610 nm. (d, h, l, p, t) Overlay images. Scale bar = 25 μm. The excitation wavelength was 405 nm. Figure S13: Cytotoxicity of SiRh evaluated on HeLa cells by MTT assay. Error bars represent standard deviation from triplicate measurements. Table S1: Detailed Data for Limit of Detection (LOD).
