# Supplementary material for: Fluorescent Probe with Dual Energy Transfer Process for Selective Fe3+ Detection and Cellular Imaging
Source: Micromachines (Basel). 2026 Aug 13;17(8):957. doi: 10.3390/mi17080957 (PMC13515341; doi:10.3390/mi17080957)
Supplement: Supplementary file 1 [file micromachines-17-00957-s001.zip › micromachines-4395044-supplementary.pdf]

Supporting information

Fluorescent Probe with Dual Energy Transfer Process for Selective Fe^{3+} Detection and Cellular Imaging

Lei Zhong †, Liang Chen †, Fagu Zeng, Xiuji Wang * and Yihua Gao *

School of Pharmacy, Guangdong Medical University, Dongguan 523808, China
* Correspondence: yxywxj@gdmu.edu.cn (X.W.); gaoyh@gdmu.edu.cn (Y.G.)
† These authors contributed equally to this work.

1. Experimental

Apparatus and materials

Chemical reagents purchased from Aladdin Scientific Corp. (Shanghai, China) were analytical pure and used without further purification. All the solvents used in spectroscopic study were of HPLC grade. Milli-Q water was used as deionized water. NMR spectra were recorded on a 600 MHz Bruker Ascend Evo 600 spectrometer (Bruker Corporation, Billerica, MA, USA). HRMS and ESI-MS spectra were obtained on an Agilent 6500 LC-MS spectrometer (Agilent Technologies Inc., Santa Clara, CA, USA) and a Shimadzu LCMS-8045 spectrometer (Shimadzu Corporation, Kyoto, Japan), respectively. UV-vis absorption and fluorescence spectra were collected on a Shimadzu UV-3101PC spectrophotometer (Shimadzu Corporation, Kyoto, Japan) and a Shimadzu RF-5301PC spectrophotometer (Shimadzu Corporation, Kyoto, Japan), respectively. Cell images were acquired with a Leica DMi8 confocal laser scanning microscope (Leica Microsystems GmbH, Wetzlar, Germany) equipped with a ×63 objective lens. NMR spectra were processed using TopSpin (5.0). Chemical structures were drawn using InDraw (7.0). Data fitting and figure export were carried out in OriginPro (2024). Manuscript layout and tables were prepared with WPS Office (2026).

2. Structure characterization

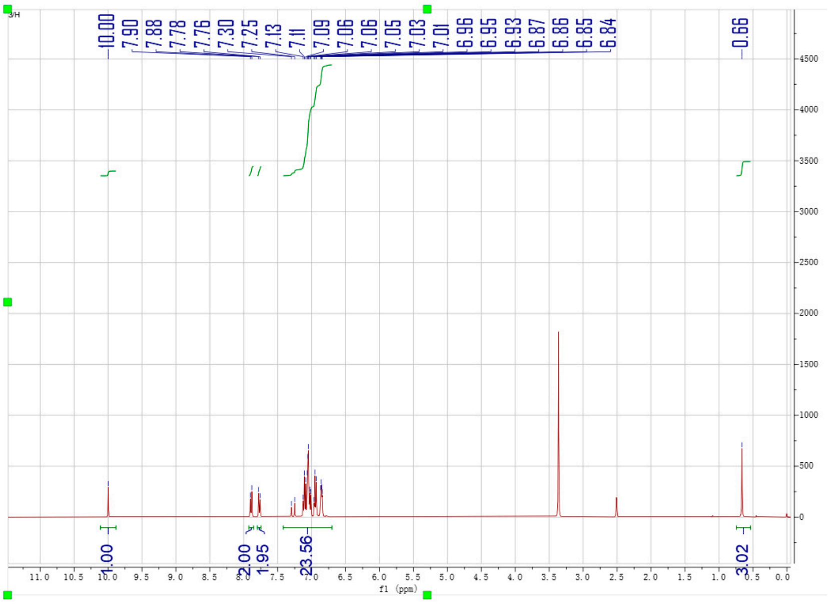

Figure S1. ^{1}H NMR of compound **SiloleCHO** (600 MHz, DMSO).

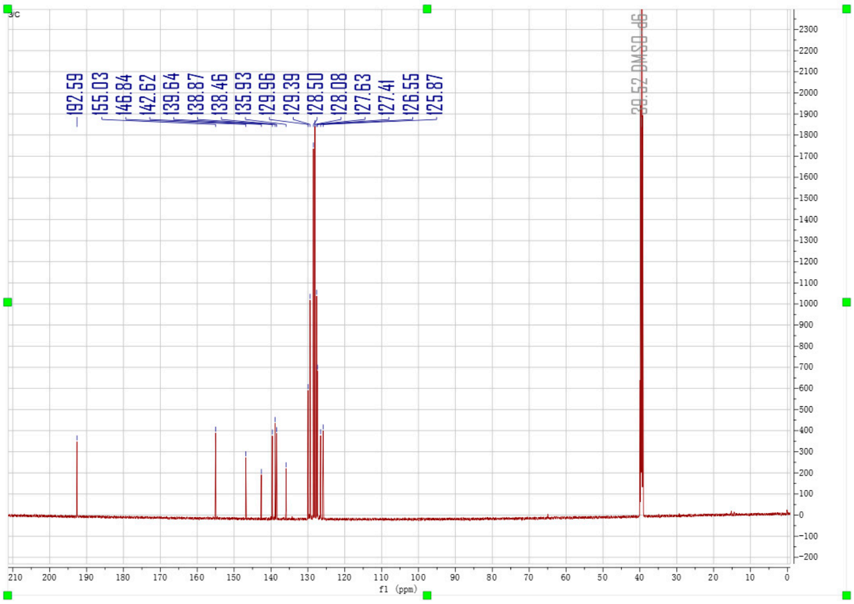

Figure S2. ^{13}C NMR of compound **SiloleCHO** (150 MHz, DMSO-d6).

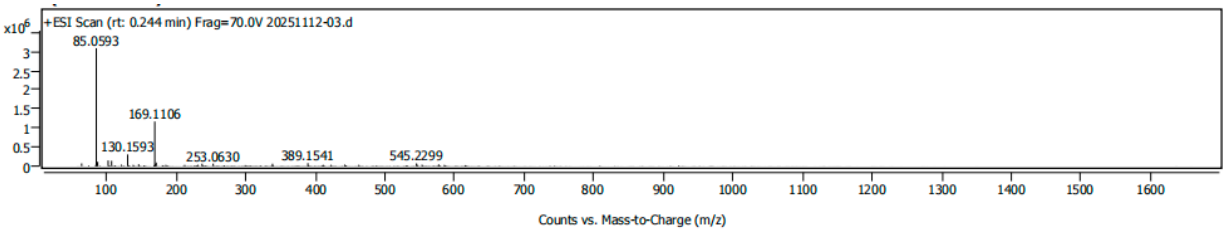

Figure S3. ESI-MS spectra of **SiloleCHO**.

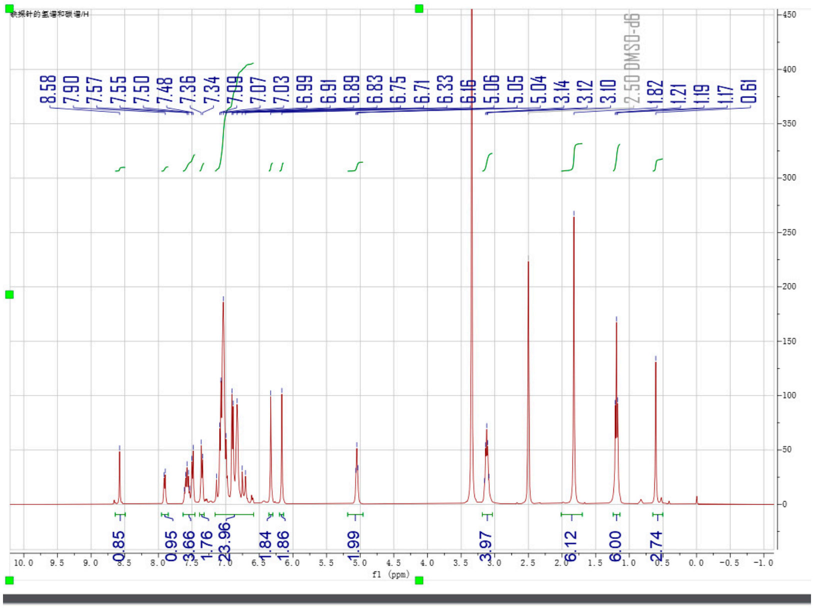

Figure S4. ^{1}H NMR of compound **SiRh** (600 MHz, DMSO-d6).

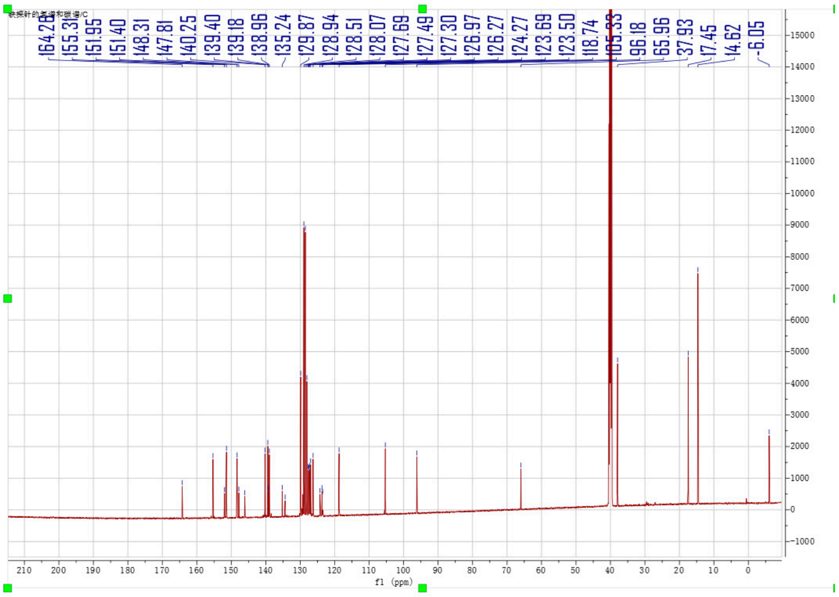

Figure S5. ^{13}C NMR of compound **SiRh** (150 MHz, DMSO-d6).

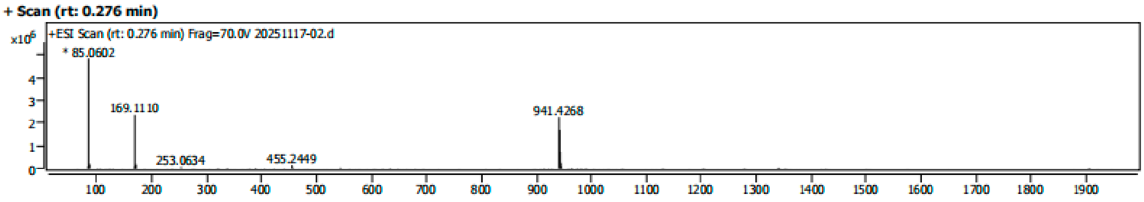

Figure S6. HRMS spectra of **SiRh**.

3. Additional spectra and images

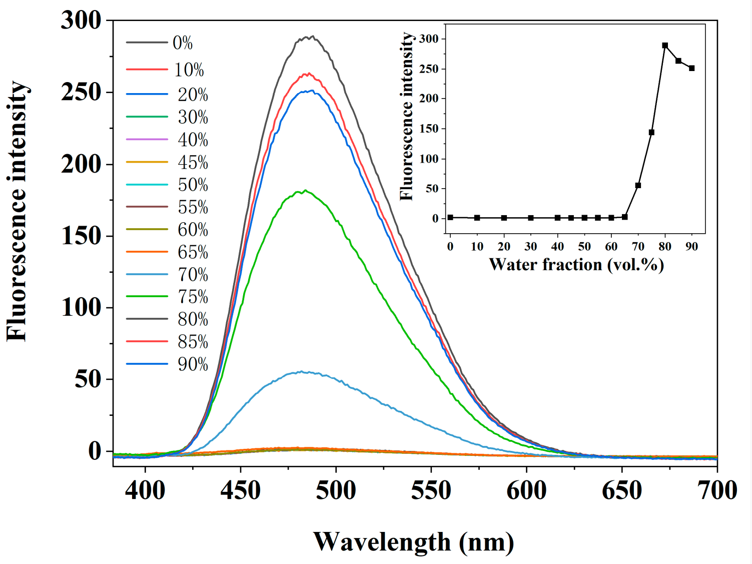

Figure S7. Fluorescence spectra of **SiRh** (10 μM) in THF/H_2O mixtures with different water fractions. Excitation was performed at 360 nm. Inset: Fluorescence intensity at 480 nm as a function of water fraction.

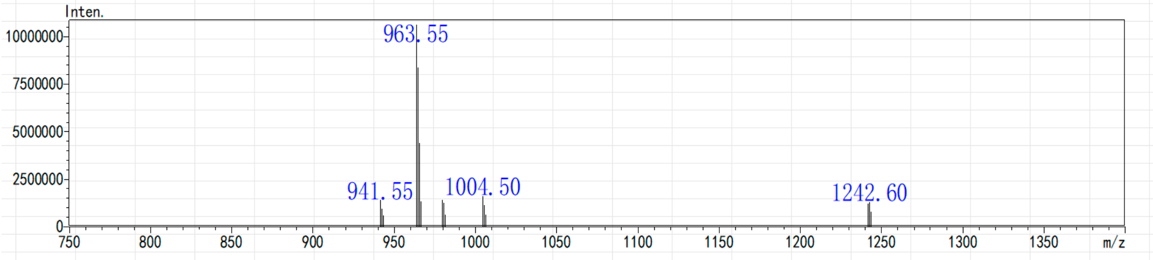

Figure S8. ESI-MS spectra of the mixture of **SiRh** (10 μM) and Fe^{3+} (20 μM) in THF/H_2O solution (2/8, v/v).

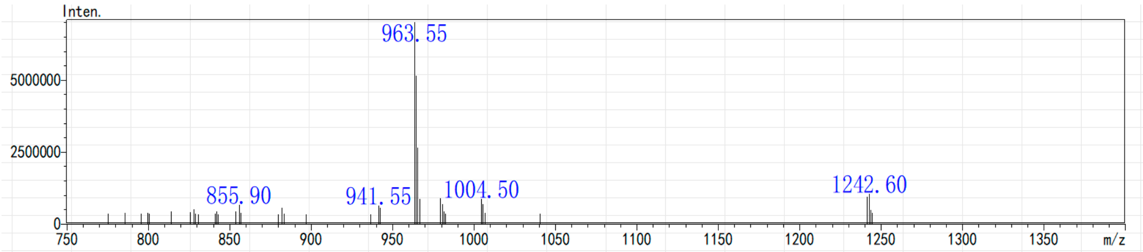

Figure S9. ESI-MS spectra of the mixture of **SiRh** (10 μM) and Fe^{3+} (20 μM) in THF/H_2O (8:2, v/v).

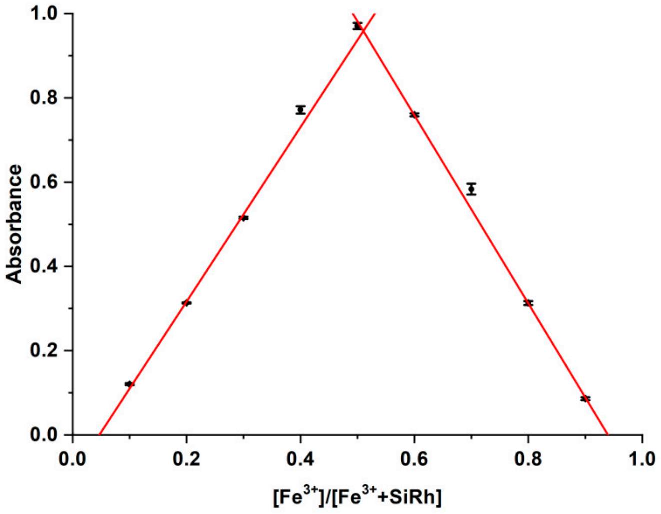

Figure S10. Job's plot of **SiRh** and Fe^{3+} in THF/H_2O solution (8/2, v/v). The total concentration of **SiRh** and Fe^{3+} was 50 μM. Detection wavelength was set at 530 nm.

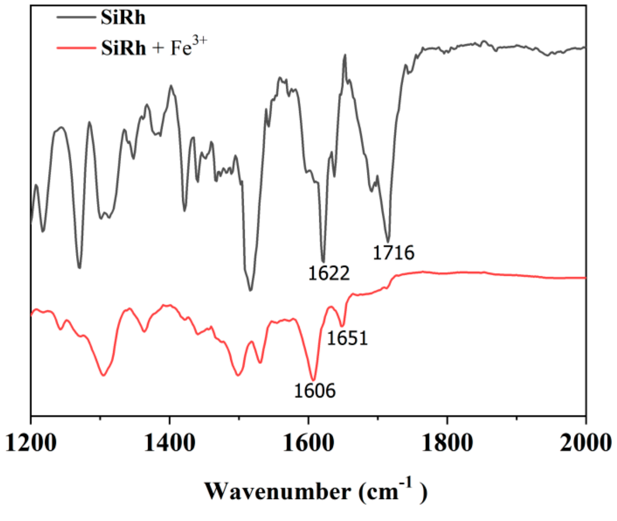

Figure S11. Partial IR spectra of **SiRh** in the absence and presence of Fe^{3+}.

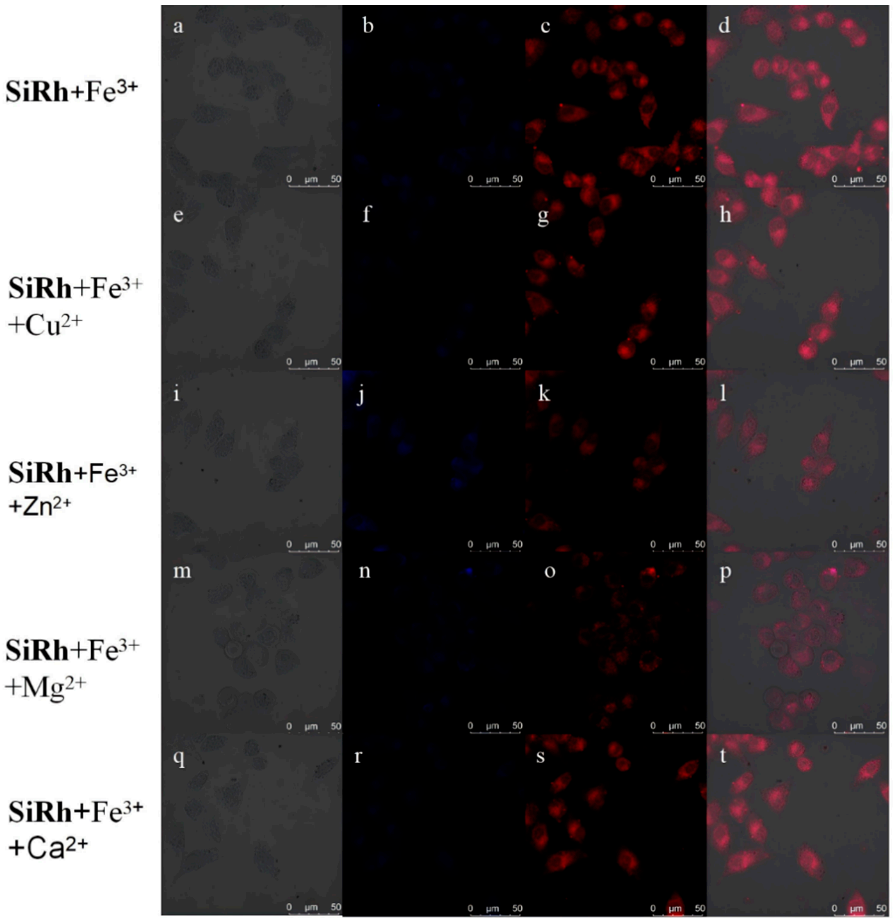

Figure S12. Fluorescence images of HeLa cells incubated with **SiRh** (10 μM) for 1h followed by 20 min of incubation with Fe^{3+} (20 μM) in the absence and presence of coexisting ions (Cu^{2+}, Zn^{2+}, 200 μM; Mg^{2+}, Ca^{2+}, 1 uM). (a, e, i, m, q) Bright field images. (b, f, j, n, r) Blue channel images: 450-510 nm. (c, g, k, o, s) Red channel images: 550-610 nm. (d, h, l, p, t) Overlay images. Scale bar = 25 μm. The excitation wavelength was 405 nm.

4. Cell viability test

Cells were seeded into 96-well plates at a density of 5,000 cells per well. A stock solution of the probe was prepared in DMSO and the final DMSO concentration in the cellular experiments was below 0.1% (v/v). Following 12 h of incubation, the cells were treated with the probe at various concentrations. After an additional 24 h incubation, 10 μL of MTT solution (5 mg/mL) was added to each well, and the cells were incubated for another 4 h. Subsequently, 100 μL of SDS-HCl solution (10% SDS, 0.01 M HCl) was added to each well. After 6 h of incubation at 37 °C, cell viability was determined by measuring the absorbance at 570 nm.

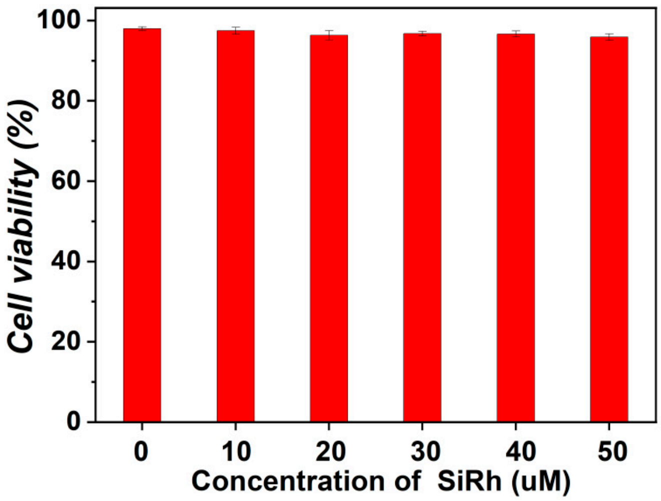

Figure S13. Cytotoxicity of **SiRh** evaluated on HeLa cells by MTT assay. Error bars represent standard deviation from triplicate measurements.

Table S1. Detailed Data for Limit of Detection (LOD)

Media Condition	Regression Equation	The Standard Deviation of The Blank (σ)	Slope (S)	Calculated LOD (μM)
THF/H_2O = 8/2	y=14.5431+50.42102*x	1.2682	50.42102	0.083
THF/H_2O = 2/8	y=0.45179+0.78309*x	0.0664	0.78309	0.28

The limit of detection (LOD) was calculated using the formula LOD = 3.3×(σ/S), where σ is the standard deviation of the intercept of the regression line (blank signal, n=10) and S is the slope of the calibration curve.
